# Population ageing and health financing: A method for forecasting two sides of the same coin^☆^

**DOI:** 10.1016/j.healthpol.2022.10.004

**Published:** 2022-12

**Authors:** Jonathan Cylus, Gemma Williams, Ludovico Carrino, Tomas Roubal, Sarah Barber

**Affiliations:** a European Observatory on Health Systems and Policies, Belgium; b London School of Economics, United Kingdom; c London School of Hygiene and Tropical Medicine, United Kingdom; d WHO Centre for Health Development, Japan; e University of Trieste, Italy; f Kings College London, United Kingdom; g WHO Country Office in Ukraine, Ukraine

**Keywords:** PASH, Population Ageing financial Sustainability gap for Health systems, SHI, Social Health Insurance

## Abstract

There is a perception that population ageing will have deleterious effects on future health financing sustainability. We propose a new method—the Population Ageing financial Sustainability gap for Health systems (or alternatively, the PASH)—to explore how changes in the population age mix will affect health expenditures and revenues. Using a set of six anonymized country scenarios that are based on data from countries in Europe and the Western Pacific representing a diverse range of health financing systems, we forecast the size of the ageing-attributable gap between health revenues and expenditures from 2020 to 2100 under current health financing arrangements. In the country with the largest financing gap in 2100 (country S6) the majority (87.1%) is caused by growth in health expenditures. However in countries that are heavily reliant on labour-market related social contributions to finance health care, a sizeable share of the financing gap is due to reductions in health revenues. We argue that analyses giving equal attention to both health expenditures and revenues steers decision makers towards a more balanced set of policy options to address the challenges of population ageing, ranging from targeting expenditures and utilization of services to diversifying revenue.

## Highlights

• Prior studies of health financing sustainability and ageing explore only health expenditures.
• We propose a method to forecast how ageing affects both health expenditures and revenues.
• Population ageing will put upwards pressures on health expenditures.
• Countries relying on labour market financing face declining revenues as populations age.
• There are a range of policy options to address financing gaps.

## 1 Introduction

Population ageing is one of the greatest accomplishments of the 20th century [1]. Yet, increases in longevity coupled with reductions in fertility have frequently concerned decision-makers due to the potential implications of changes in population age-mix for labour markets and the economy, as well as for the sustainability of public programs and spending, particularly the health sector. The literature defines health system fiscal sustainability as the ability of a government to maintain public spending on health at a credible and serviceable position over the long term; this has commonly been assessed based on historical and projected growth in health expenditures, and oftentimes by comparing growth in health expenditures to economic growth [2], [3], [4]. The intuition behind this approach is that growth in health care expenditure would not necessarily be problematic given unlimited resources [4], however, should spending grow faster than the economy, a sustainability issue would emerge over time [3]. As stressed by the OECD, governments need information about future health care spending and funding sources to assess their health financing sustainability [2]. Nevertheless, although the models used to forecast health care spending typically incorporate estimates of how an ageing population will increase expenditure on health services, they do not account explicitly for an ageing population's revenue impact [2]. We aim to develop a tool to fill this gap.

Given the positive association between health care utilization and increasing calendar age [5], many analysts assume population ageing will result in considerable growth in health spending. Since this expenditure tends to be sourced primarily from public funds, there is great policy interest to quantify how population ageing is likely to affect health expenditure trends. In long-term budgetary projections, the European Commission (EC) forecasts that age-demographic changes will increase public expenditure on health care in EU Member States by 1.1 percentage points of GDP from 6.8% to 7.9% on average between 2016 and 2060 [6]. However, considerable debate in the economics and health policy literature has highlighted that the role of calendar age in determining health expenditure is likely to be small compared to other demand-side factors such as the role of time-to-death, particularly the final year of life, as well as individual characteristics such as morbidity [7]. Moreover, supply-side factors impact on expenditure growth, such as technological advances in health care interventions, national income growth, and the way in which health care services are organized and delivered, which influence prices and the volume of care [7], [8], [9].

In addition to its effects on expenditures, population ageing may also affect the ability to raise funds for health care systems, especially if population ageing causes declines in labour market participation and slowdowns in economic growth. Public funds for health (as well as for other government functions) are raised through a diverse set of mechanisms, including social contributions linked to the labour market, income taxes, consumption taxes, and property taxes, among other forms of taxation. The ability to generate revenues from each of these sources may vary as the population age structure in a country changes. This is confirmed based on evidence from various countries, including the United States, Japan and New Zealand, where studies have found income tax revenues decline as populations age, while consumption taxes are generally unaffected [10], [11], [12]. The effects of ageing on government revenues may be particularly notable in countries that are heavily reliant on labour markets as a revenue base [13]. A recent study in Austria found that population ageing will lead to declines in per person social security contributions and income taxes, though assumptions of high real wage growth and Austria's high reliance on taxation of pensions may counteract the adverse effects of ageing on public revenues [14].

Most analyses of the effects of population ageing on health financing focus exclusively on the effects of population ageing on health expenditures, while ignoring the effects of population ageing on health revenues [[6], [15], [16]]. By focusing solely on health expenditures, the insinuation coming from these analyses is that sustainability can only be achieved through reductions in expenditure growth. However the changing age-mix also affects the generation of revenues. Ignoring the potential impact of ageing on revenues severely limits the policy relevance of any analysis of sustainability. Indeed, even though ageing might have a small effect on future health expenditure growth, its overall impact on health finances and public budgets might be important, especially if there are significant declines in health revenues.

In this paper we propose a new method to assess the sustainability of health financing by considering effects on both health revenues and expenditures as the population age mix changes — what we refer to as the Population Ageing financial Sustainability gap for Health systems (or alternatively, the PASH). We simulate some policy options to fill the gap on the revenues side, since this has received less attention previously compared to expenditures. This provides a starting point for countries looking to prioritize their own policy response to population ageing.

We believe our simple approach is innovative and policy relevant for two main reasons. First, we extend the standard approach for assessing health financing sustainability in the context of population ageing by looking at how both health expenditures and revenues are affected by a changing population age-mix. Indeed, we introduce a tool which allows countries to explore, under current health financing arrangements, how changes in their population age-mix would result in changes in the gap between health expenditures and revenues over time.

Second, by drawing attention to the effects of ageing on both health expenditures and revenues, our proposed method provides policymakers with a broader set of policy options to consider in an effort to address financing shortfalls. Current models that focus only on the effects of ageing on expenditures naturally suggest that the most obvious solution is to slow the rate of expenditure growth. Conversely, analyses that give equal attention to both health expenditures and revenues captures a more holistic picture of the impact of population ageing on the sustainability of health financing. This is particularly important for policymakers as it highlights the need for a balanced set of policies addressing the consequences of ageing, ranging from those targeting expenditures, prices and utilization of services to those that address budget shortfalls through changes in the approach to revenue raising.

## 2 Materials and methods

To calculate the PASH, we simulate effects of population ageing on both health expenditures and revenues from 2020 to 2100. While the novelty of our approach lies in the joint focus on expenditure and revenues, our method follows the standard approach for assessing health financing sustainability in the context of population ageing: that is, we forecast how health expenditures and revenues are affected by a changing population age-mix, assuming current health financing arrangements were to remain constant. As our aim is to demonstrate the PASH methodology rather than make country-specific recommendations, we chose to apply the PASH to actual data from six countries at different stages of age demographic transition with diverse health financing arrangements (Australia, Bulgaria, Japan, Slovenia, United Kingdom and Vietnam), but we refer to them throughout the paper as anonymized country scenarios (S1-S6). The six anonymized country scenarios are described in Table 1. All analyses use population projections by five-year age groups (from 0-4 to 85 years and older) from the United Nations Population Division [17].

**Table 1:** Descriptive statistics for selected country scenarios.

	S1	S2	S3	S4	S5	S6
**Age demographics**						
% Population 65+ in 2020	15%	17%	28%	11%	18%	8%
% Population 65+ in 2060	23%	21%	38%	15%	26%	24%
% Population 65+ in 2100	27%	19%	36%	14%	29%	29%
						
**Health financing (in % of public health spending)**						
Taxes on income, profits, and capital gains	63%	12%	24%	3%	35%	4%
Social contributions	0%	54%	55%	91%	19%	87%
Taxes on property	10%	1%	6%	0%	13%	0%
Taxes on goods and services	27%	32%	15%	6%	33%	8%
Percent of SHI funds from general tax revenues	N/A	33%	45%	4%	N/A	13%
Average per person health spending for a 70-74 year old (relative to a 20-24 y.o.)	4.7	5.6	6.8	4.5	4.0	8.7
Domestic general government health spending per person (2018 $US PPP adjusted)	3,457	942	3,787	2,287	3,631	201
Domestic general government health spending (% of GDP, 2018)	6.4	4.2	9.2	6.0	7.9	2.7

Source: [[1], [18], [19], [20], [21], [22], [28], [29]].

Note: Tax data are from 2017 for all countries except S6, which is 2016. GDP = Gross Domestic Product.

### 2.1 Health expenditures and population ageing

Simulating the contribution of population ageing to future health expenditure patterns requires data on per person health expenditures by age. For each scenario we used available country health expenditure by age data for the latest available year [18], [19], [20], [21]); these data generally include only public expenditures though for the country scenario based on Japan, out-of-pocket expenditures are also included. Where the data were disaggregated by single year of age, we calculated average per person spending by 5-year age groupings to align with the corresponding population data.

We multiply per person health expenditures for each age group by the respective 5-year age group population in each year and sum the expenditure across all age groups. Dividing the resulting sum by the total population size in each year gives a measure of per person health expenditures which varies over time due only to changes in the population age structure. This allows us to isolate the contribution of changes in population age structure to health expenditure growth over time. In taking this approach, the assumption is that relative per person spending patterns by age remain constant in the future. This assumption implies that future changes in other established drivers of health spending patterns, i.e., prices, technology, or entitlements, will have similar effects on expenditures across all age groups. A decade of health expenditure by age data from EU countries confirms that this is a plausible assumption [5].

### 2.2 Health revenues and population ageing

Estimating how health revenues will be affected by population ageing requires data on per person tax and social contributions by age; unfortunately these data are not produced regularly for most countries. In lieu of country-specific data, we use National Transfer Accounts (NTAs) data for six countries which represent different stages of demographic transition and different models of public financing: Italy (2008), Japan (2004), Sweden (2003), Thailand (2006), Uruguay (2006) and USA (2011). These countries provide plausible data which we then use to construct illustrative synthetic age-profiles of per person social contributions, taxes on income, taxes on goods and services, and taxes on property. We calculate forecasts of per person health revenues for each anonymized country-scenario in three steps.

First, as the synthetic age-profiles of public sector revenues are based on combined data from countries with very differently sized public sectors, to make them more comparable, we normalize the original values from the NTAs (expressed in dollars per capita) by dividing each type of revenues for each age group by the level of per person taxes on goods and services among the age group 45 to 49 years for each country. We then average the country-age-specific normalized values to obtain synthetic revenue profiles by age and by type of revenue source, illustrated in the Online Appendix Figure A2.

Second, we take the synthetic per person revenues by 5-year age group profiles and multiply by the 5-year age group population in each year for each of our 6 anonymized country scenarios and sum across all age groups. Dividing the sum by total population for each year for the respective country scenario gives a measure of per person revenues for each source of taxation and social contributions which varies from year-to-year only because of changes in the population age structure.

Third, in order to provide a measure of total per person health revenues (rather than total per person government revenues), we aggregate the revenues from different sources into a single measure, using appropriate weights. We do this by weighting our revenue projections to reflect the health financing mix in the base year. This is based on the percentage of SHI revenues coming from social contributions for each anonymized country scenario which is available in the WHO Global Health Expenditure Database [22] and then within the remaining general tax transfers, these are disaggregated to reflect the country scenario's public revenue mix overall, available from the OECD [23]. As with the expenditure projections, our assumption is that although the age-mix of the population will change in the future, the relative levels of each source of public revenue that are generated per person by age group will remain constant, allowing us to isolate the effects of population ageing on health revenues. We discuss the limitations of the data used and the method to calculate health revenues in the discussion section.

The difference between the per person expenditure and revenue forecasts, referred to as the PASH, provides an estimate of the financial sustainability gap due to ageing, conditional on the current approach to health financing and service delivery being maintained in the future.

## 3 Results

Table 1 contains descriptive statistics for all six anonymized country scenarios. S3 is expected to have more than one-third of its population 65 years and older for most of the century. S6 is expected to have the most dramatic increase in its share of the population 65 years and older, rising from 8% in 2020 to 29% by 2100. The country scenarios have a wide range of health financing systems. S1 is financed predominantly through general tax revenues (and thus its revenue structure largely mirrors that of its public revenues overall); the other countries are financed through a mix of social insurance contributions and general taxes. S5 relies on some social contributions though it is primarily financed through general tax revenues. S4 is almost exclusively financed through social contributions, whereas in S3 almost half of its SHI funds come from general taxation.

The health expenditure by age data also reveal important cross-country differences. The average 70 to 74 year old in the S5 consumed 4.0 times as much health care as the average 20-24 year old in 2018. In comparison, in S6, the relative differential was 8.7.

**Fig. 1:**
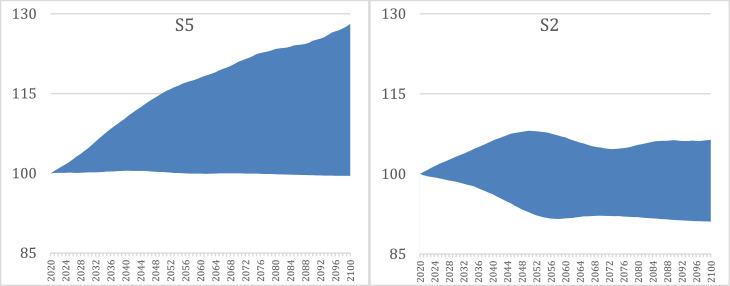
Revenues, expenditures, and the population ageing financial sustainability gap for health systems (PASH), S5 and S2, 2020-2100 (index 2020 = 100).

To illustrate the PASH method, Fig. 1 presents health revenue and expenditure projections for two of the country scenarios with distinct health financing approaches: S2, a typical social health insurance system, and S5, a typical tax financed system. The upper boundary of the blue shaded area in each panel represents the expenditure projection, and the lower boundary represents the revenue projection; both are indexed to be equal to 100 in 2020, the base year (i.e. assuming revenues=expenditures in the base year). The blue shaded areas represent the financing gap (i.e. the PASH) between revenues and expenditures. For S5 (left panel), ageing is expected to result in a sizeable increase in health expenditure per person in relative terms by the end of the projection period of 28.1% relative to 2020 (upper boundary of the blue shaded area). This increase corresponds to an additional 0.3% average annual growth per person in health expenditure attributable to the changing age-mix over the projection period. Based on the current financing regime in S5, ageing is not expected to have any material effect on revenue generation (lower boundary of the blue shaded area), with around a 0.5% decrease in health revenues per person by 2100 due to ageing. In 2100, S5 is therefore projected to have a PASH of 28.6 base points, amounting to a $1,038 per person financing gap in 2018 PPPs or 2.2% of GDP based on the latest available domestic general government health expenditure data for 2018.

Alternatively, in S2 (right panel), ageing is anticipated to have a fairly minor impact on health expenditure trends. The effects of ageing on per person health expenditures are forecast to peak in 2050 at 8.1% above 2020 levels. By 2100, health spending is expected to be just 6.4% higher than in 2020 as a result of population ageing. However, on the revenue side the effects are comparatively more pronounced, with around 8.9% lower health revenues per person expected due to changes in age demographics in 2100 than in 2020. This means that while the projected PASH in S2 in 2100 of 15.3 base points ($145 per person in 2018 PPPs or 0.6% of GDP) is smaller than that of S5, in S2, declines in revenue are projected to be responsible for 58.2% of the PASH at the beginning of the next century, whereas in S5 nearly all (98.3%) of the PASH in 2100 is attributable to increases in expenditures per person.

**Fig. 2:**
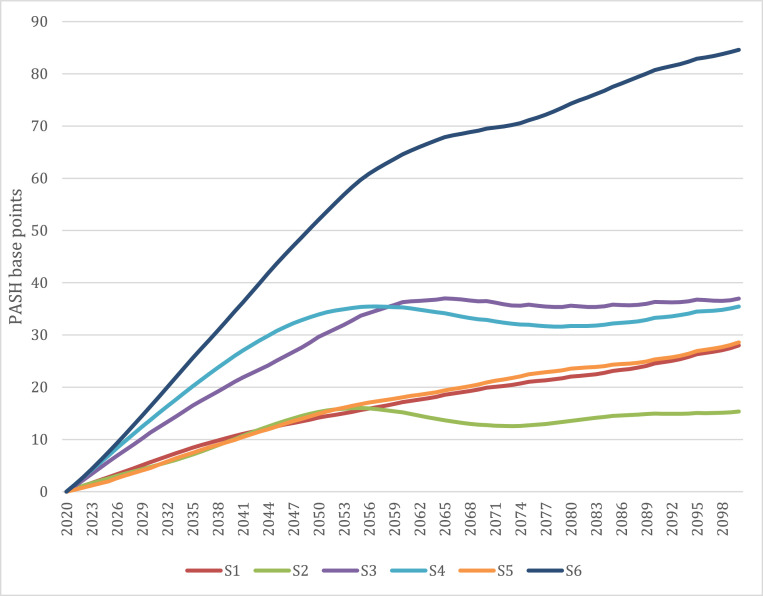
Population ageing financial sustainability gap for health systems (PASH), 2020-2100, selected countries.

Fig. 2 shows the PASH for all six country scenarios between 2020 and 2100. The largest gap by 2100 is expected to be in S6, where the gap between revenues and expenditure is expected to increase by 84.6 base points compared to 2020, or alternatively, a gap of around $170 per person in 2018 PPPs or 2.3% of GDP; 87.1% of this increase is due to expected growth in expenditures. However, in S4, where the gap is forecast to reach 35.4 base points, in line with the forecast for S3, nearly half of the gap (44.2%) is due to reductions in revenue generation. In S3, 28.7% of the gap is due to declining revenues. In S1, population ageing is expected to result in increases in per person revenues over the forecast period so that 111.0% of the PASH in 2100 is due to growth in health expenditures.

There are many policy options countries can take to address their ageing health financing sustainability gap. To better understand the potential role of revenue raising mechanisms specifically, we simulate the PASH using S4’s population by age and health expenditure by age data, but we incorporate health revenue raising regimes from each of the other five country scenarios. Although imposing one country's revenue raising approach on another country is politically and practically infeasible, the aim is to illustrate the implications of different country revenue raising approaches using a single country's population age-mix.

**Fig. 3:**
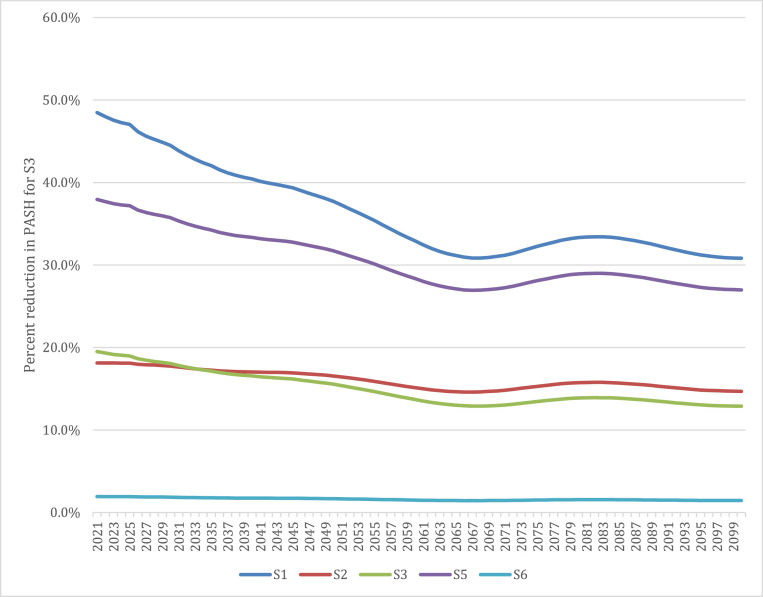
Share of PASH reduced using the revenue raising approach of selected countries, S4 population and health expenditure by age profile.

Fig. 3 shows the percentage of the PASH that could be reduced in each year if S4 were to use a different country scenario's health revenue raising approach. Relying primarily on general taxation, as in S1 or S5, would reduce S4’s PASH by approximately 1/3^rd^ by the end of the century. This amounts to approximately $705 or $617 per person in 2018 PPPs (1.9% or 1. 6% of GDP, respectively) based on S4 domestic general government health expenditure levels in 2018. S2 and S3’s comparatively more mixed financing approaches as compared to S4 would still reduce the financing gap by 14.7% and 12.9%, respectively.

## 4 Discussion

This paper presents a refined methodology to monitor health system financial sustainability in the context of population ageing. While there are many other initiatives to forecast how the changing age structure can influence future health expenditure trends, there have been insufficient efforts to date to couple those analyses with health revenue forecasts. Monitoring the contemporaneous effects of population ageing on health system revenues and expenditures has important advantages from a policy perspective.

First, it provides a more realistic picture of the implications for health financing of population ageing. For example, looking only at the effects of population ageing on expenditures, it would appear that a country like S2 has no notable health system financial sustainability issues to be concerned with as its population ages. However due to its high reliance on labour market financing for its health system, the PASH approach demonstrates that population ageing is likely to result in significant challenges without structural changes to the way health revenues are raised in that country. Additionally, S6 is expected to have a fairly substantial PASH through the rest of the century. While much of this is due to increased expenditure resulting from its notable transition in its age-structure over the next 80 years, shifts in the mix of health revenues towards more general tax financing could help to make up some of the gap.

Second and most importantly, the PASH analysis highlights the wide range of policy options available to manage health financing as the population ages. Focusing exclusively on health expenditure forecasts when assessing the effects of population ageing may lead policymakers to put in place fiscal rules to impose constraints on expenditure growth over the long-term without similarly considering the availability of revenues in tandem [24] or to otherwise search for other more blunt policy measures that slow expenditure growth quickly, such as reducing entitlements. This may be an appealing policy response for some decision-makers given that other actions that can help reduce growth in health spending, such as strengthening prevention and promoting healthy ageing, can take a long time to show demonstrable savings [25]. However, there are other policy options which become visible as a result of the PASH approach.

For example, expected increases in health spending due to ageing may be addressed in part by changing the approach to revenue generation. Health financing systems that are heavily reliant on labour markets as a revenue base are the most susceptible to population ageing due to reductions in the traditional working-age population over the projection period. Important policy options therefore include increasing labour market participation at older ages or requiring adequate contributions from older persons towards health [26]. For example, in Japan, since 2008 people over age 75 have had their health system contributions paid automatically from their pensions towards the late stage-medical care system [27]. In the context of S2, the analysis from the PASH shows that the current financing approach whereby more than half of health care expenditure is funded by labour-related contributions, will likely need to change in the future as the population ages. Alternative revenue raising mechanism, such as increases in taxation or social contribution rates may be more sustainable to finance projected age-related increases in expenditure, though such changes in taxation rates may be distortionary.

Likewise, many countries could benefit by diversifying revenue sources to rely more on those sources which are less adversely affected by population ageing, or those which in some countries may even increase as the age-structure shifts. In S6, for example, despite expected declines in social contributions due to population ageing, all three other forms of taxation that we review in this study are expected to increase due to changes in population age structure between 2020 and 2100. While there are many countries that continue to rely on labour market financing to fund their health systems, our analyses show that as populations age, labour markets are likely to be inadequate as a sustainable revenue base. Rather, a general tax-based financing would help to address financial sustainability challenges.

An important policy message is therefore that addressing health financing related challenges brought on by population ageing requires action not only by health systems themselves, but also by finance policymakers who have more immediate control over the design of tax policies and revenue generation mechanisms. This is made much more apparent through the inclusion of both expenditures and revenues in the analysis of financial sustainability.

Our analysis has assumed that the current composition of health revenues would be maintained. Hypothetically, if in the future years a government would face a decline in the current sources of revenues, this could be compensated by, e.g., reallocating revenues previously destined to other services, or issuing public debt. We believe that either of these choices would have to be carefully considered by any government, as they embed important consequence for the provision of public services and the intertemporal sustainability of public debt. We believe that by simulating future budget gaps we can inform governments as per the likelihood that any of these (or any other) budgetary change will have to be made in order to balance the health budget. Moreover, our analysis has further shown that past policy decisions on the health-revenue base can shape the impact of ageing on sustainability. While this is not advocating for a sudden change in revenue policies, we believe policy circles should critically examine the potential weaknesses in a country health system sustainability as determined by the choices made in structuring the revenue mix.

There are a number of important limitations to this analysis. As mentioned, we model the effects of changes in the population age mix holding expenditure by age and revenue by age profiles constant. This enables us to isolate the effects of population ageing, however it is important to note that these expenditure and revenue age-profiles could change in the future. In future analysis it may be useful to incorporate more dynamic expenditure and revenue age-profiles, although doing so would arguably reflect inter complex interplay of a range of factors, not just age-mix-specific effects. Additionally, we have made use of illustrative tax revenue by age profiles that are based on cross-country averages. To operationalize this type of analysis going forward, country-specific data on tax revenues by age would be most useful. Likewise, we have made assumptions about the current mix of health financing for our country scenarios based on internationally comparable data; it is possible that the exact mix of revenues in country health systems differs somewhat from what can be ascertained from these sources. We also assume that health revenues are equal to expenditures in the base year, which may not be accurate, as expenditures can be financed from reserves or borrowing. Future research may wish to relax this and other assumptions of the analysis. Moreover, to conduct this type of analysis in a wider range of countries requires greater availability of health spending by age data than there is currently. Finally, we acknowledge that other supply-side factors besides ageing impact on health expenditure and revenues, such as technological advances in health care interventions and national income growth [7], [8], [9]. While these factors are not accounted for in our study, we hope that future research could incorporate these and other factors into the PASH approach to obtain a more holistic picture.

## 5 Conclusion

Population ageing is occurring in all countries, but it does not present inevitable or unsolvable challenges for health systems. The way decision-makers respond to it is highly dependent on the way its effects are measured. Our simulation models used for the PASH methodology demonstrate that comparative analyses that give equal attention to both health expenditures and revenues provide a more balanced set of policy options for addressing the health financing consequences of ageing. The options range from targeting expenditures and utilization of services to diversifying revenue generation. For some countries, making changes now in how health revenues are raised may result in more money for health as the population age-mix changes in the future, as shown from countries like S1 and the S5 that rely heavily on general tax revenues to finance health. While there is a widely held perception that population ageing will have deleterious effects on health financing, this is ultimately a function of how financing systems are designed.

We declare no conflicts of interest.

## Appendix Supplementary materials

Image, application 1
