# Supplementary material for: Population ageing and health financing: A method for forecasting two sides of the same coin
Source: Health Policy. 2022 Dec;126(12):1226–32. doi: 10.1016/j.healthpol.2022.10.004 (PMC9709572; doi:10.1016/j.healthpol.2022.10.004)
Supplement: Supplementary file 1 [file mmc1.docx]

Online Appendix, Figure A1, normalised government revenues from different sources, by contributors age.


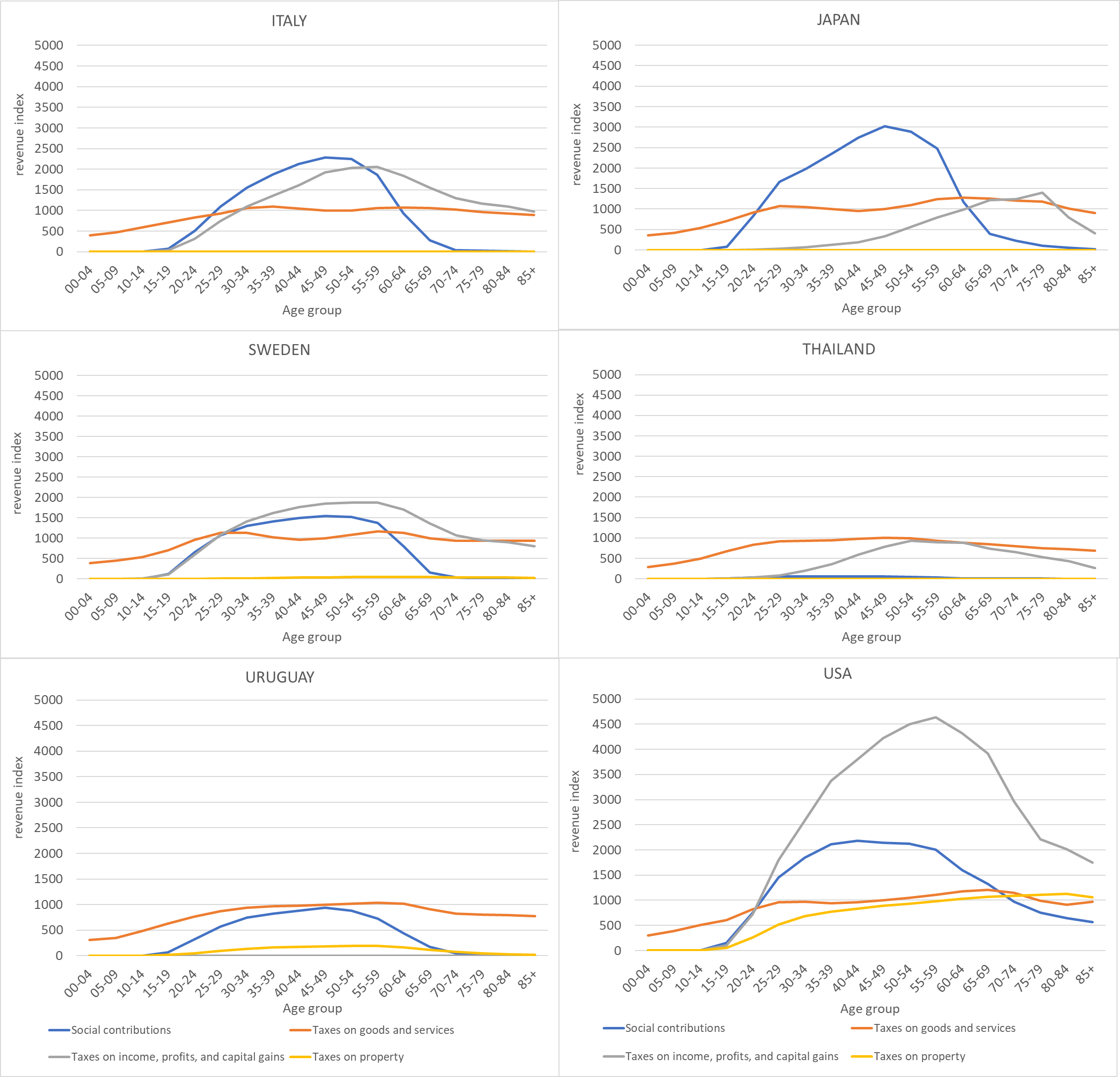


*Note: we show the normalized revenue profiles for Italy, USA, S3, Sweden, Uruguay, Thailand. These profiles have then been used to generate the synthetic revenue profile in the PASH. The Figure A1 suggests that revenue profiles show many similarities and some differences across countries, which may represent different approaches to public policy and budgeting. Social contributions and taxes on income are among the largest source of public revenues in all countries except USA and especially Thailand, and exhibit a concave relationship with age, with the maximum revenues being provided by middle-aged groups. Exceptions are S3, where the tax-revenue curve has its peak at older ages, and Thailand, where social contributions revenues are limited. Revenues from taxes on goods and services have a much flatter distribution across age groups, and are relatively more consistent over the life course in Sweden, Uruguay and Thailand.*

*Online Appendix, figure A2. Per person synthetic health revenues by source, by age*

Source: (13)
